# GPC3 reduces cell proliferation in renal carcinoma cell lines

**DOI:** 10.1186/1471-2407-14-631

**Published:** 2014-08-29

**Authors:** Marina Curado Valsechi, Ana Beatriz Bortolozo Oliveira, André Luis Giacometti Conceição, Bruna Stuqui, Natalia Maria Candido, Paola Jocelan Scarin Provazzi, Luiza Ferreira de Araújo, Wilson Araújo Silva, Marilia de Freitas Calmon, Paula Rahal

**Affiliations:** Department of Biology, Instituto de Biociências, Letras e Ciências Exatas - IBILCE/UNESP, Rua Cristóvão Colombo, 2265, 15054-000 São José do Rio Preto, SP Brazil; Department of Genetics, University of São Paulo, and Center for Integrative Systems Biology (CISBi-NAP/USP), Av. Bandeirantes, 14049-900 Ribeirão Preto - São Paulo, Brazil

**Keywords:** GPC3, Cell lines, Cell proliferation, Renal carcinoma, Transfection

## Abstract

**Background:**

Glypican 3 (GPC3) is a member of the family of glypican heparan sulfate proteoglycans (HSPGs). The *GPC3* gene may play a role in controlling cell migration, negatively regulating cell growth and inducing apoptosis. *GPC3* is downregulated in several cancers, which can result in uncontrolled cell growth and can also contribute to the malignant phenotype of some tumors. The purpose of this study was to analyze the mechanism of action of the *GPC3* gene in clear cell renal cell carcinoma.

**Methods:**

Five clear cell renal cell carcinoma cell lines and carcinoma samples were used to analyze *GPC3* mRNA expression (qRT-PCR). Then, representative cell lines, one primary renal carcinoma (786-O) and one metastatic renal carcinoma (ACHN), were chosen to carry out functional studies. We constructed a *GPC3* expression vector and transfected the renal carcinoma cell lines, 786-O and ACHN. *GPC3* overexpression was analyzed using qRT-PCR and immunocytochemistry. We evaluated cell proliferation using MTT and colony formation assays. Flow cytometry was used to evaluate apoptosis and perform cell cycle analyses.

**Results:**

We observed that *GPC3* is downregulated in clear cell renal cell carcinoma samples and cell lines compared with normal renal samples. *GPC3* mRNA expression and protein levels in 786-O and ACHN cell lines increased after transfection with the *GPC3* expression construct, and the cell proliferation rate decreased in both cell lines following overexpression of GPC3. Further, apoptosis was not induced in the renal cell carcinoma cell lines overexpressing GPC3, and there was an increase in the cell population during the G1 phase in the cell cycle.

**Conclusion:**

We suggest that the *GPC3* gene reduces the rate of cell proliferation through cell cycle arrest during the G1 phase in renal cell carcinoma.

## Background

Renal cell carcinoma (RCC) is the most lethal urological disease [1] and is responsible for 3% of all malignant neoplasms [2]. The incidence of RCC has been increasing over the last few decades [3] due to advances in early detection of renal tumors provided by ultrasound, computed tomography and magnetic resonance imaging [4–6].

RCC is a heterogeneous histological disease, and clear cell renal cell carcinoma (CCRCC) is the most common histological subtype, making up approximately 75-80% of the cases of renal tumors [1, 7]. Renal cell carcinoma is diagnosed in the advanced stage of the disease in 25% of patients [8]. Although nephrectomy and radiotherapy are effective, 30% of patients develop metastatic disease after treatment, with a median survival period of one year [7, 9].

The occurrence of RCC is usually sporadic, although genetic syndromes can cause a familial pattern of inheritance. For example, Von–Hippel Lindau disease, which is associated with mutations and inactivation of the *VHL* gene [10], is correlated with the occurrence of clear cell renal cell carcinoma [11]. Therefore, it is important to identify genes associated with CCRCC and to better understand their possible mechanisms of action in renal tumor cells. Several studies have identified genes differentially expressed in clear cell renal cell carcinoma and normal renal samples [9, 12]. One of these genes is *GPC3*, which is decreased in clear cell renal cell carcinoma [9].

*Glypican 3* (*GPC3*), which is located on the human X chromosome (Xq26), is a member of the heparan sulfate proteoglycan (HSPG) family [13, 14]. This protein can bind to the surface of the cell membrane via glycosylphosphatidylinositol (GPI) anchorage [15]. *GPC3* plays important roles in cell growth regulation, proliferation, differentiation, migration and apoptosis [16, 17]. It is differentially expressed in some tumor types – in hepatocellular carcinoma and melanoma, *GPC3* is highly expressed [18]; however, its expression is reduced in ovarian and breast cancer [19, 20], a finding which suggests that *GPC3* may be involved in tumor development [21]. The *GPC3* gene is considered a potential molecular marker in hepatocellular carcinoma [22] and may act as a tumor suppressor in the ovary [19].

In the present study, we investigated the mechanisms of action of *GPC3* in renal cell carcinoma using colony formation, cell proliferation, cell cycle progression and apoptosis assays to assess the potential role of *GPC3* in this type of cancer.

## Methods

### Clear cell renal cell carcinoma samples

Thirty-five clear cell renal cell carcinoma samples and two normal renal fresh-frozen tissue samples were obtained from the Tumor Bank from the Pio XII Foundation/IBILCE-UNESP, Sao Paulo, Brazil. The use of patient-derived material was approved by the Research Ethics Committee of the Tumor Bank from the Pio XII Foundation/IBILCE-UNESP, Sao Paulo, Brazil, and written consent was obtained from all patients. Tissues were obtained during surgery on patients undergoing tumor resection, and the diagnosis of clear cell renal cell carcinoma was verified post-operatively using histopathology. The samples were classified according to the criteria provided by the International Union against Cancer [23].

### Cell lines

The cell lines ACHN, 786-O, A-498, CaKi-1 and CaKi-2 were obtained from American Type Culture Collection (ATCC, Manassas, VA, USA). ACHN and A-498 cells were cultured in a MEM Alpha medium (Gibco by Life Technologies, Grand Island, NY, USA), CaKi-1 and CaKi-2 cells were cultured in a McCoy’s 5A medium (Gibco by Life Technologies, Grand Island, NY, USA) and 786-O cells were cultured in a RPMI1640 medium (Gibco by Life Technologies, Grand Island, NY, USA). Cell lines were supplemented with 10% FBS (Cultilab, SP, Brazil), 100 U/mL penicillin (Invitrogen, Grand Island, NY, USA) and 100 μg/mL streptomycin (Invitrogen, Grand Island, NY, USA) and were grown in a 37°C, 5% CO_2_ atmosphere.

*GPC3* mRNA expression was analyzed in all cell lines. Then, representative cell lines, one primary renal carcinoma (786-O) and one metastatic renal carcinoma (ACHN), were chosen to carry out the functional studies.

### Plasmid construction

DNA oligonucleotides were chemically synthesized, and appropriate restriction sites were introduced via PCR amplification with the following primers: CATCGGTACCATGGCCGGGACCGTGCG (Forward) and TCGACTCGAGCACCAGGAAGAAGAAGCACACCACCG (Reverse). After PCR purification, products and the pcDNA3.1/V5-HisB vector were digested by the restriction enzymes KpnI and XhoI (Uniscience, New England Biolabs, Hitchin, UK). The products were ligated by T4 DNA ligase (Uniscience, New England Biolabs, Hitchin, UK). The construct was confirmed using DNA sequencing.

### Transfection

The pcDNA3.1/*GPC3* expression vector and pcDNA3.1 (empty vector) were transfected into ACHN and 786-O cell lines using Lipofectamine 2000 (Invitrogen, Carlsbad, CA, USA) according to the manufacturer’s manual.

### RNA extraction and qRT-PCR

Total RNA was extracted using TRIzol reagent (Life Technologies, Grand Island, NY, USA) according to the manufacturer’s instructions. Approximately 5 μg of total RNA from each sample was used to synthesize cDNA, using the High Capacity cDNA Kit (Applied Biosystems, Foster City, CA, USA) according to the manufacturer’s instructions. Real Time PCR was performed using an ABI Prism 7300 Real Time PCR system and SYBR Green PCR Core Reagent (Applied Biosystems, Warrington, UK) following the manufacturer’s protocol. The primer sequences were designed using Primer 3 software: *GPC3*: GTGCTTTGCCTGGCTACATC (Forward) and TCCACGAGTTCTTGTCCATTC (Reverse), and *GAPDH* (endogenous control): ACCCACTCCTCCACCTTTGA (Forward) and CTGTTGCTGTAGCCAAATTCGT (Reverse). In brief, the reaction mixture (20 μL total volume) contained 25 ng of cDNA, gene-specific forward and reverse primers for each gene and 10 μL of 2× Quantitative SYBR Green PCR Master Mix. The samples were tested in triplicate.

The relative expression of each specific gene was calculated using the following formula: R = (E target)^ΔCt target (control - sample)^/(E endogenous)^ΔCt endogenous (control - sample)^, which had been published previously [24]; a cutoff higher than a 2-fold change was used. The expression of the gene *GPC3* was analyzed in thirty-five clear cell renal cell carcinoma samples and the cell lines ACHN, 786-O, A-498, CaKi-1 and CaKi-2. Two normal renal fresh-frozen tissue samples were used as the normal reference (control group). All samples were collected from the renal cortex.

### Immunocytochemistry

ACHN and 786-O cells were seeded on coverslips in 24-well plates. The cells were washed with PBS twice and fixed with 4% paraformaldehyde for 30 min. Endogenous peroxidase activity was blocked with 3% hydrogen peroxide in methanol for 30 min in the dark and, after washing in PBS, the non-specific proteins were blocked in 1% bovine serum albumin (BSA) for 1 h. The cells were incubated at 4°C overnight with rabbit polyclonal anti-GPC3 (5 μg/mL) (ABCAM, Cambridge, UK) diluted in 1% BSA. After washing, cells were incubated with the biotinylated secondary antibody (1:200) (Santa Cruz Biotechnology, California, USA), diluted in 1% BSA for 45 min at 37°C and then exposed to an HRP-conjugated streptavidin complex (Santa Cruz Biotechnology, CA, USA). The reactions were visualized using DAB substrate (Dako, Cambridge, UK) and the slides were counterstained with hematoxylin. Densitometric analyses of GPC3 were performed with an Axioshop II Microscope (Zeiss, Germany) using the Software Axiovision (Zeiss). For the analyses, eleven different fields from the coverslips were used and 15 points were analyzed. The values were obtained on an arbitrary scale.

### Colony formation assay

ACHN and 786-O cells transfected with pcDNA3.1/*GPC3* and pcDNA3.1 were plated in 6-well plates (300 μL cell per well) containing 700 μg/mL geneticin (G418, Sigma Aldrich, St Louis, MO, USA). After 14 days, the colonies were stained with 0.01% crystal violet. Each experiment was performed in triplicate and in two independent assays.

### Proliferation assay

ACHN and 786-O were seeded into 96-well plates. After the transfection, 1 mg/mL 3-(4,5-dimethylthiazol-2-yl)-2, 5-diphenyl-tetrazolium bromide (MTT) (Sigma Aldrich, St Louis, MO, USA) was added to the wells and incubated for 30 min at 37°C. Then, the MTT was removed, 100 μL of 100% DMSO (Sigma Aldrich, St Louis, MO, USA) was added to each well and the absorbance was measured at 562 nm. Each experiment was performed in triplicate and in two independent assays.

### Apoptosis assay

Apoptotic cells were analyzed using a FITC Annexin V Apoptosis Detection Kit II (BD Biosciences, San Diego, CA, USA) according to the manufacturer’s instructions. After transfection, cells were washed twice with PBS and then resuspended in binding buffer. Next, 5 μL FITC-Annexin V and 5 μL Propidium Iodide (PI) were added and the cells were incubated for 15 min in the dark at room temperature. The cells were analyzed using an easyCyte 5-HT flow cytometry (Millipore Guava Technologies, Hayward, USA). Data are from two independent experiments.

### Cell cycle analysis

ACHN and 786-O cells were analyzed 24 h, 48 h and 72 h after the transfection. The cells were washed twice with PBS and then fixed with ice-cold ethanol (70%). Next, the samples were stained with 200 μL of Guava Cell Cycle Reagent (EMD Millipore Corporation, Hayward, CA, USA), incubated for 30 min at room temperature, and the analysis was conducted by using the easyCyte 5-HT flow cytometry (Millipore Guava Technologies, Hayward, USA). Two independent experiments were performed.

### Statistical analysis

Statistical analysis was performed using GraphPad Prism 5 Software. The Mann–Whitney U Test and Wilcoxon Single Ranks Test were used to compare the protein expression levels detected through immunohistochemistry. The comparisons of protein expression levels in cells overexpressing GPC3 to cells lacking GPC3 were performed using analysis of variance (ANOVA), with the appropriate post-hoc test. Group comparisons in the MTT assay were performed with two-tailed paired Student’s *t* test. In all analyses, the differences were considered statistically significant when p < 0.05.

## Results

### Analysis of *GPC3*gene expression in clear cell renal cell carcinoma samples and renal carcinoma cell lines

**Figure 1:**
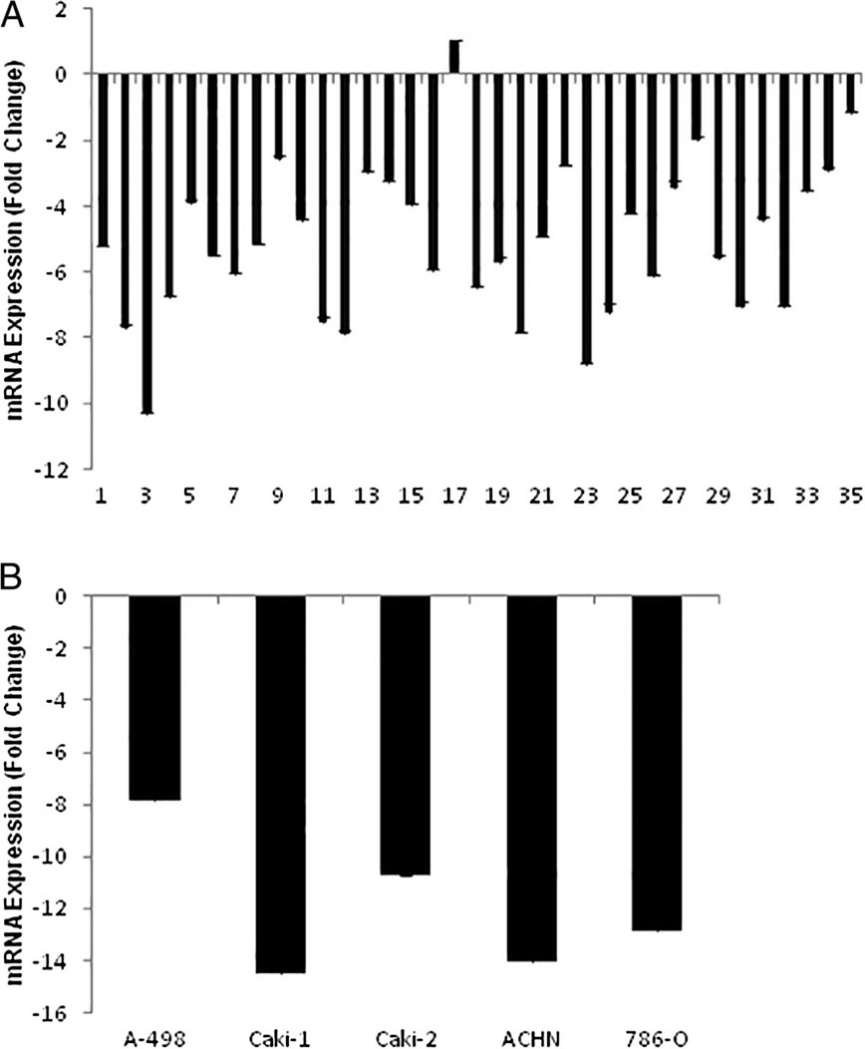
**Endogenous** ***GPC3*** **expression in clear cell renal cell carcinoma.** Quantitative mRNA expression of *GPC3* was determined via qRT-PCR in clear cell renal cell carcinoma and renal carcinoma cell lines and shown as fold change (log2) relative to expression in normal renal tissue. **A)** Expression (mRNA) of *GPC3* in 35 clear cell renal cell carcinoma samples. **B)** Expression (mRNA) of *GPC3* in five clear cell renal cell carcinoma cell lines.

The *GPC3* gene was downregulated in all clear cell renal cell carcinoma samples, with the exception of one (Figure 1A). There was no association between *GPC3* gene expression and the clinical data of the clear cell renal cell carcinoma patients (Table 1). The renal carcinoma cell lines were also compared with normal renal samples, with fold-change values for gene expression ranging from -1 to -10.3 in primary clear cell renal cell carcinoma samples and -7.8 to -14.4 in primary renal carcinoma cell lines (CaKi-2, A-498 and 786-O) and metastatic renal carcinoma cell lines (CaKi-1 and ACHN) (Figure 1B). *GPC3* expression was lower in metastatic cell lines than in primary cell lines. The same observation was made in the case of clear cell renal cell carcinoma samples, in which *GPC3* gene expression was lower in metastatic samples when compared with non-metastatic samples. Unfortunately, the number of metastatic clear cell renal cell carcinoma samples used in this study was too small to perform a statistical test. To assess the potential role of *GPC3* in this type of cancer, we performed assays in the 786-O and ACHN renal carcinoma cell lines.

**Table 1:** **Description of clinical data of patients with clear cell renal cell carcinoma**

Variable	Number of patients
**Gender**	
Male	19
Female	16
**T stage**	
T1	13
T2	14
T3	7
T4	1
**N stage**	
N0	31
N1	2
N2	1
N3	1
**M stage**	
M0	28
M1	7
**Average age in years**	
Males	57.8
Females	57.1
**Smoker**	
No	30
Yes	5
**Alcoholic**	
No	27
Yes	8

**Figure 2:**
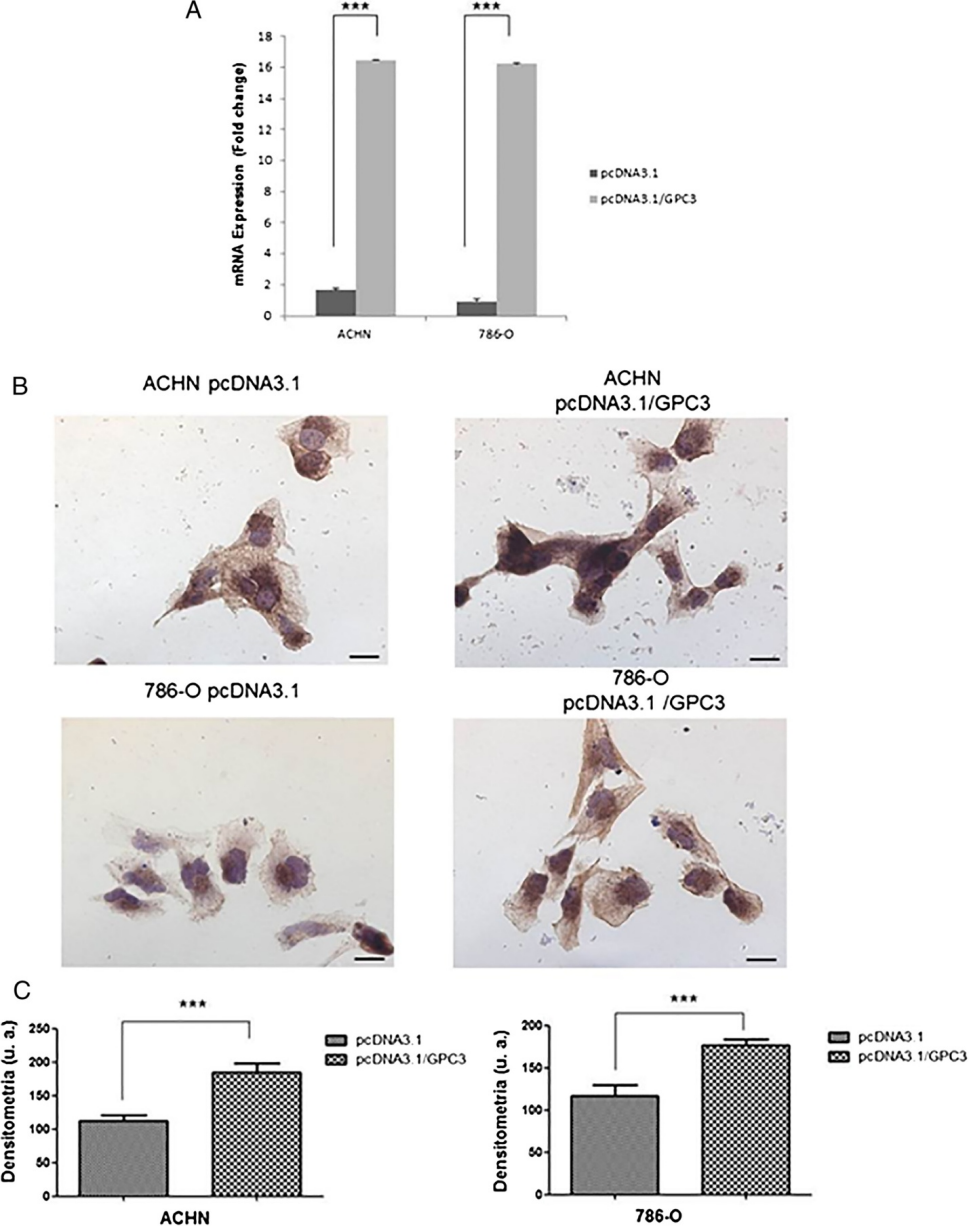
***GPC3*** **expression in renal cell carcinoma cell lines.** Restoration of *GPC3* expression after transfection with the plasmid pcDNA3.1/*GPC3*. ACHN and 786-O cells were transiently transfected with pcDNA3.1 (empty vector) or pcDNA3.1/*GPC3* and re-expression of *GPC3* was confirmed 48 h post-transfection by qRT-PCR and immunocytochemistry. **A)** Quantitative mRNA expression of the *GPC3* gene in renal cell carcinoma cell lines after 48 h of transfection with the pcDNA3.1/*GPC3* plasmid is shown as fold change (log2) relative to expression of *GPC3* in cell lines after 48 h of transfection with pcDNA3.1 (empty vector). **B)** Immunolocalization of the GPC3 protein in ACHN and 786-O cell lines after 48 h of transfection with the plasmids pcDNA3.1-GPC3 and pcDNA3.1 (empty vector). Bars = 20 μm. **C)** Densitometry graphic of GPC3 in ACHN and 786-O cell lines (***p < 0.0001, Mann Whitney test).

We evaluated *GPC3* mRNA and protein expression in the ACHN and 786-O cell lines before and after transfection with the pcDNA3.1/*GPC3* expression vector or an empty vector using qRT-PCR and immunohistochemistry, respectively. *GPC3* was upregulated in both cell lines 48 h after transfection with the pcDNA3.1/*GPC3* vector (Figure 2A). GPC3 protein expression was increased in the cells transfected with pcDNA3.1/*GPC3* vector compared with cells transfected with an empty vector in both cell lines. GPC3 immunostaining increased significantly in the membranes of ACHN and 786-O cells transfected with the pcDNA3.1/*GPC3* vector (p < 0.0001) (Figures 2B and C).

### GPC3 suppresses colony formation

**Figure 3:**
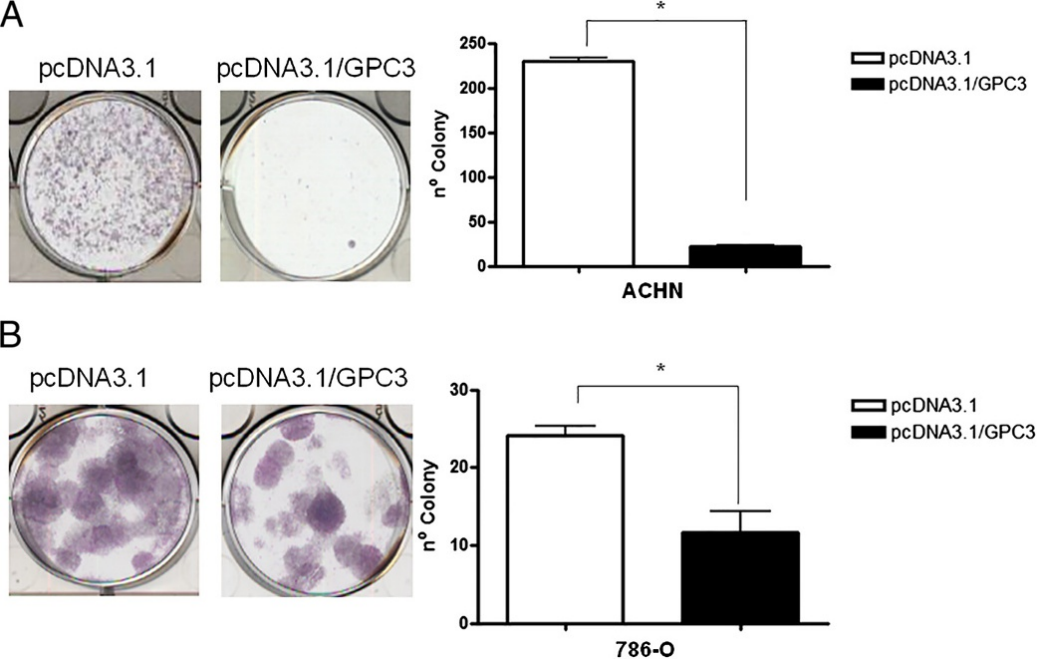
**Effect of GPC3 on colony formation.** The *GPC3* gene suppresses the colony formation in ACHN **(A)** and 786-O **(B)** cell lines. Tumor cell proliferation was assessed *in vitro*. Cells were transiently transfected with pcDNA3.1 (empty vector) or pcDNA3.1/*GPC3* and replated 24 h post-transfection for selection with Geneticin/G418. After 14 days of selection, colonies were stained with Giemsa and counted. Data are presented as the mean of two independent experiments ± SEM. Group comparisons were carried out using Student’s *t* test, *p < 0.01.

The pcDNA3.1/*GPC3* and pcDNA3.1 vectors were transfected in ACHN and 786-O cell lines, and colony formation ability was assessed after 14 days. Cell lines overexpressing GPC3 had suppressed growth in the colony formation assay. *GPC3*-transfected cells grew significantly fewer colonies than cells transfected with an empty vector in both cell lines (p < 0.01) (Figure 3).

### Effect of GPC3 on proliferation in cell lines

**Figure 4:**
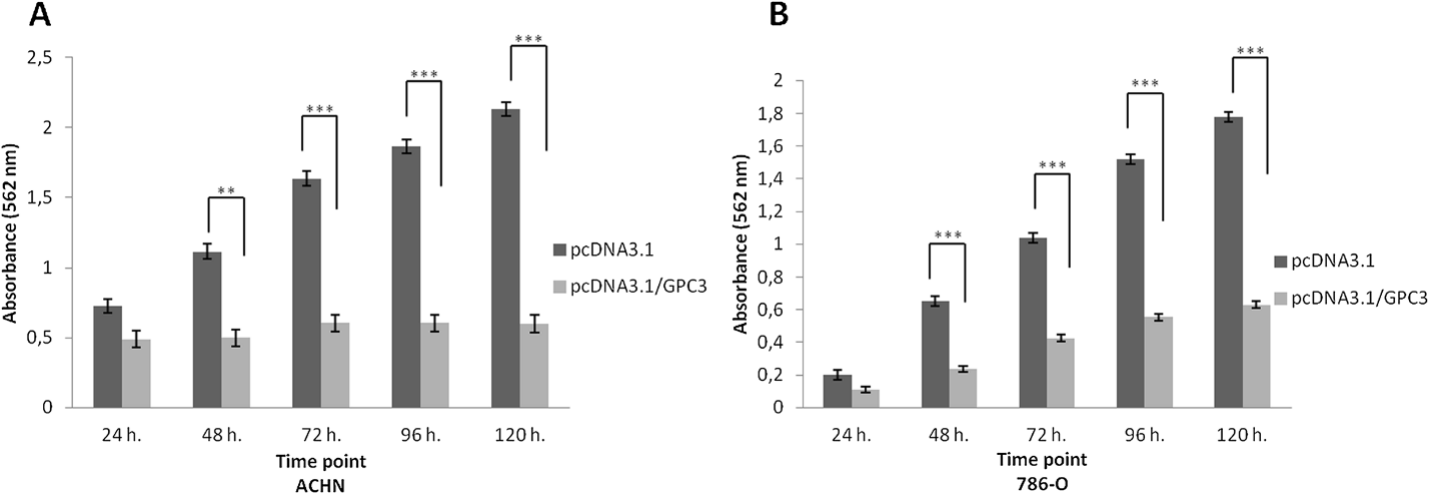
**Effect of GPC3 on cell proliferation.** Cell viability of ACHN and 786-O cells was measured 24 h, 48 h, 72 h, 96 h and 120 h post-transfection with pcDNA3.1 (empty vector) and pcDNA3.1*/GPC3* by MTT assay. Data are presented as the mean of two independent experiments ± SEM. Group comparisons were carried out using Student’s *t* test. **A)** ACHN cells overexpressing GPC3 experienced a significant reduction in proliferation rate after 48 h (**p < 0.01), 72, 96 and 120 h (***p < 0.0001). **B)** 786-O overexpressing GPC3 experienced a significant reduction in proliferation rate after 48 h, 72 h, 96 h and 120 h (*** p <0.0001).

Cell proliferation was determined by an MTT assay in ACHN and 786-O cells overexpressing GPC3 at 24 h, 48 h, 72 h, 96 h and 120 h after transfection. ACHN cells overexpressing GPC3 experienced a significant reduction in cell proliferation compared with ACHN cells lacking GPC3 expression after 48 h (p < 0.01), 72 h, 96 h and 120 h (p < 0.0001) (Figure 4A). 786-O cells overexpressing GPC3 also had reduced proliferation after 48 h, 72 h, 96 h and 120 h (p < 0.0001) (Figure 4B).

### Effect of GPC3 on apoptosis in cell lines

**Figure 5:**
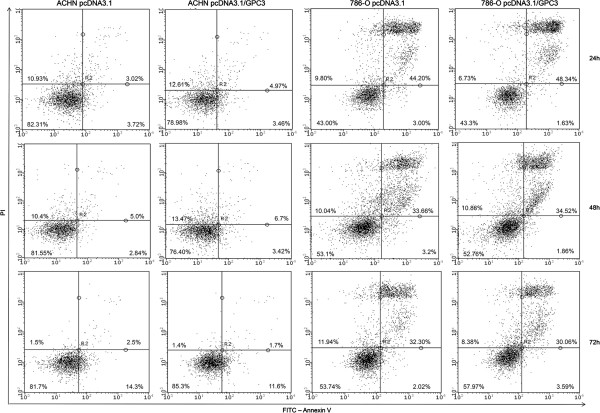
**The effect of GPC3 on apoptosis.** Apoptosis in ACHN and 786-O cell lines was analyzed using flow cytometry 24 h, 48 h and 72 h post-transfection. No difference in apoptosis was observed between ACHN and 786-O cell overexpressing and lacking GPC3 at any of the time points analyzed. Viable cells (Annexin V-/PI-) are located in the bottom left, early apoptotic cells (Annexin V+/PI-) in the bottom right, late apoptotic cells (Annexin V+/PI+) in the top right and necrotic cells (Annexin V-/ PI+) in the top left quadrants, respectively.

The ability of GPC3 to induce apoptosis was also evaluated. The rate of apoptosis was analyzed in cell lines after 24 h, 48 h and 72 h of transfection with pcDNA3.1/*GPC3* and pcDNA3.1 vectors using FITC-Annexin V/PI. No difference in apoptosis was observed between ACHN and 786-O cells overexpressing or lacking GPC3 at any analyzed period of time (p > 0.05) (Figure 5).

### GPC3 alters cell cycle progression

**Figure 6:**
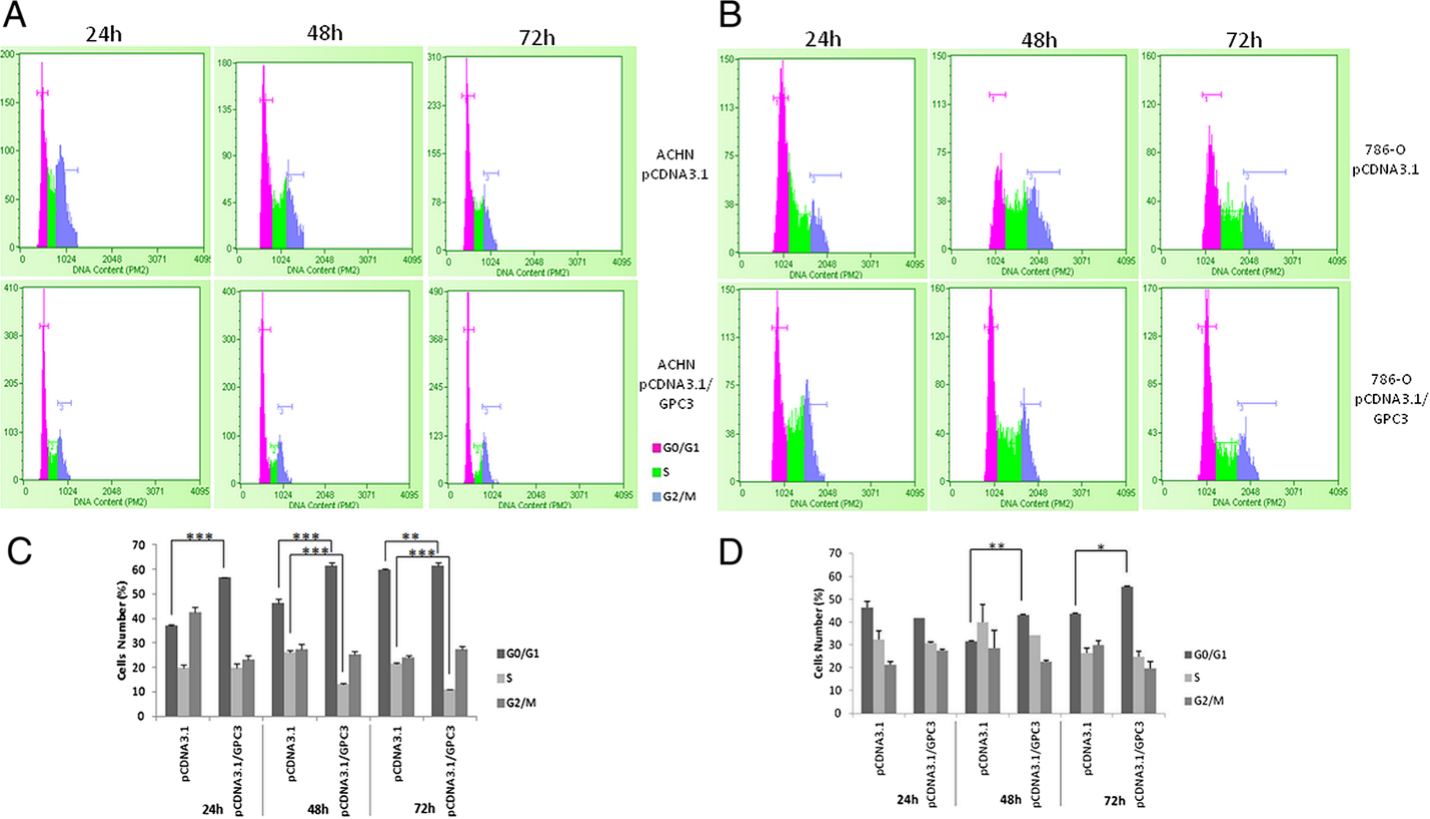
**Effect of GPC3 on cell cycle in cell lines. A)** ACHN cell cycle histogram. **B)** 786-O cell cycle histogram. **C)** The number of ACHN pcDNA3.1/*GPC3* cells was significantly increased during the G1 phase (24 h and 48 h ***p < 0.001, 72 h **p < 0.01, ANOVA) and decreased during the S phase (48 h and 72 h ***p < 0.001, ANOVA). **D)** The number of 786-O pcDNA3.1/*GPC3* cells was significantly increased in the G1 phase 48 h (**p < 0.01, ANOVA) and 72 h (*p < 0.05, ANOVA) post-transfection.

We then performed cell cycle analysis on ACHN and 786-O cell lines, using flow cytometry 24 h, 48 h and 72 h after transfection with pcDNA3.1/*GPC3*. The number of ACHN and 786-O cells overexpressing GPC3 was evaluated in each phase of the cell cycle and was compared with the number of ACHN and 786-O cells lacking GPC3. There were more ACHN cells overexpressing GPC3 in the G1 phase (24 h and 48 h, p < 0.001; 72 h, p < 0.01) and fewer cells overexpressing GPC3 in the S phase after transfection (48 h and 72 h, p < 0.001) (Figure 6A). In the 786-O cell line, a higher number of cells overexpressing GPC3 was observed in the G1 phase after transfection (48 h, p < 0.01; 72 h, p < 0.05) compared with 786-O cells lacking GPC3 (Figure 6B).

## Discussion

*Glypican-3* (*GPC3*) is a member of the family of heparan sulfate proteoglycans [25, 26]. Heparan sulfate proteoglycans (HSPGs) have profound effects on both tumor cell growth kinetics and metastasis formation [27]. HSPGs have the functional capacity to regulate a myriad of molecular interactions that induce tumor cell proliferation and metastasis [28]. Glypican-3 expression is higher in several tissues, such as the gastrointestinal tract, in human embryos [29].

In different types of tumors, GPC3 expression and its carcinogenic role are variable. In some tissues, such as ovary [19, 30], breast [20, 31] and lung (adenocarcinoma) [32], this glypican is downregulated, acting as a tumor suppressor, whereas in other tumors, it is overexpressed and functions as an oncoprotein, as observed in liver [33–35], lung (squamous cell carcinoma) [32, 36], melanoma [18] and embryonal tumors [37]. In a recent study, Gailey and Bellizzi [38] analyzed GPC3 protein expression in squamous cell carcinoma (SCCs) of diverse anatomic sites and in invasive urothelial carcinomas from the urinary bladder. They observed GPC3 staining was present in 17.3% of 156 tumors, including those of the anus (10.0%), cervix (27.3%), esophagus (28.6%), larynx (30.0%), lung (50.0%), tongue base/tonsil (12.5%), urinary bladder (12.2%), ventral tongue/floor of mouth (12.5%) and vagina (40.0%).

In the present study, *GPC3* expression was downregulated in primary clear cell renal cell carcinoma samples and cell lines. To the best of our knowledge, this report is the first that has detected downregulation of the *GPC3* gene in clear cell renal cell carcinoma cell lines. Okon et al. [39] have shown a point of distinction between GPC3 expression in chromophobe carcinoma and clear cell renal cell carcinoma; GPC3 expression was upregulated in chromophobe carcinoma and downregulated in clear cell carcinoma, agreeing with our expression results in tissue samples and cell lines.

Gailey and Bellizzi [38] also observed an absence of GPC3 expression in squamous cell carcinomas (SCCs) from the penis, skin and vulva. As opposed to the results of this study in clear cell renal cell carcinoma, GPC3 overexpression has emerged as a positive marker in liver cancer because it is highly expressed in 70-100% of hepatocellular carcinomas (HCCs) but not in normal adult liver tissue [33–35]. In addition to being proposed as a marker for liver tumor diagnosis, GPC3 has also been evaluated as a target for antibody- and cell-based therapies of HCC [18].

Cell growth was reduced in cells overexpressing GPC3 protein, as measured by colony formation and proliferation rates 48 h post-transfection in both the 786-O and ACHN cell lines used in this study. Lin et al. [19] and Murthy et al. [40] found that *GPC3* re-expression in ovarian cancer cell lines resulted in inhibition of the efficiency of colony formation. Some studies have shown that *GPC3* plays an important role in cell growth and differentiation [41, 42], such as in the case of hepatocellular carcinoma [43]. The data obtained in the present study suggest that GPC3 inhibits cell proliferation in clear cell renal cell carcinoma.

Therefore, we studied whether the reduced proliferation rate in cells overexpressing GPC3 occurs through the induction of apoptosis or through cell cycle arrest. Apoptosis, or programmed cell death, is a crucial point in the carcinogenic process. Cancer cells can overcome the apoptosis mechanism, and tumor progression continues [26, 44]. We observed that most cells, whether overexpressing or lacking GPC3, were viable at all time points in ACHN and 786-O cell lines. Our results suggest that cells overexpressing GPC3 in renal cell carcinoma do not induce apoptosis; therefore, we hypothesized that the inhibition of cell proliferation in renal carcinoma cells might occur due to cell cycle arrest.

To verify whether GPC3 overexpression arrests the cell cycle in renal cell carcinoma, we used flow cytometry to perform cell cycle analysis. The observed growth-repressive effect of GPC3 overexpressing cells was reflected by their arrest in the G1 cell cycle phase, which caused these cells to proliferate less in both cell lines. In this study, we observed changes in cell proliferation through the use of MTT assays in the ACHN cell line 48 h post-transfection. Using flow cytometry, we were able to identify changes in the cell cycle and, consequently, in cell proliferation 24 h after transfection for the same cell line. This difference is likely due to the increased sensitivity of the flow cytometry methodology to detecting changes in cell cycle and proliferation compared with the MTT assay. One previous study demonstrated that inhibiting GPC3 expression released hepatocyte carcinoma cells from G1 arrest and thus modulated cell cycle progression in this type of cancer [45].

Moreover, cell surface HSPGs inhibit invasion by promoting tight cell–cell and cell–extracellular matrix (ECM) adhesion. Previous studies documented diminished quantity and quality of heparan sulfate isolated from transformed cells compared with normal cells [46]. This alteration in heparan sulfate accompanies a reduction in the adhesive capacity of transformed cells. Low levels of cell surface heparan sulfates also correlate with high metastatic activity in melanomas [47, 48]. Furthermore, *GPC3* downregulation was more significant in invasive areas, a result which further supports an inhibitory role for *GPC3* in tumor progression in hepatocellular carcinomas [49]. Enhanced glypican-3 expression differentiates the majority of hepatocellular carcinomas from benign hepatic disorders. Conversely, glypican-3 expression is decreased in human breast cancers, and ectopic expression of GPC3 inhibits growth of breast cancer cell lines [20]. Additionally, glypican-3 was associated with the inhibition of invasion and metastasis of a mammary carcinoma cell line *in vivo* [50]. Thus, cells with normal epithelial morphology exhibit retention of cell surface HSPGs and tight attachment to the extracellular matrix. Cells that are beginning to invade exhibit reduced adhesion to the ECM, loss of epithelial morphology, and diminished levels of HSPG expression, whereas deeply invading cells completely lose HSPG expression [28].

## Conclusions

*GPC3* gene expression is downregulated in primary clear cell renal cell carcinoma, and GPC3 protein overexpression in clear cell renal cell carcinoma cell lines arrests cells during the G1 phase of the cell cycle, consequently reducing the proliferation rate. These results suggest that GPC3 acts as a tumor suppressor in clear cell renal cell carcinoma.

## Abbreviations

GPC3: Glypican 3
HSPG: Heparan sulfate proteoglycan
qRT-PCR: Real time quantitative polymerase chain reaction
MTT: 3-(4,5-dimethylthiazol-2-yl)-2,5-diphenyltetrazolium bromide
mRNA: Messenger ribonucleic acid
RCC: Renal cell carcinoma
CCRCC: Clear cell renal cell carcinoma
VHL: Von Hippel-Lindau tumor suppressor
GPI: Glycosylphosphatidylinositol
ATCC: American Type Culture Collection
FBS: Fetal bovine serum
PCR: Polymerase chain reaction
RNA: Ribonucleic acid
cDNA: Complementary deoxyribonucleic acid
GAPDH: Glyceraldehyde-3-phosphate dehydrogenase
HRP: Horseradish peroxidase
3: 3′-diaminobenzidine
FITC: Fluorescein isothiocyanate
PI: Propidium iodide
PBS: Phosphate buffered saline
ECM: Extracellular matrix.
